# Framing the conversation: use of PRECIS-2 ratings to advance understanding of pragmatic trial design domains

**DOI:** 10.1186/s13063-017-2267-y

**Published:** 2017-11-10

**Authors:** Paula Darby Lipman, Kirsty Loudon, Leanora Dluzak, Rachael Moloney, Donna Messner, Catherine M. Stoney

**Affiliations:** 1 Westat, 1600 Research Boulevard, Rockville, MD 20850 USA; 2 NMAHP Research Unit, Unit 13 Scion House, Stirling University Innovation Park, Stirling, FK9 4NF UK; 3 Center for Medical Technology Policy, World Trade Center Baltimore, 401 East Pratt Street, Suite 631, Baltimore, MD 21202 USA; 4 National Heart, Lung, and Blood Institute (NHLBI), National Institutes of Health (NIH), RKL2, BG RM 10220, 6701 Rockledge Drive, Bethesda, MD 20817 USA

**Keywords:** Pragmatic trials, Trial design, Effectiveness trials, Mixed methods, PRECIS-2 tool

## Abstract

**Background:**

There continues to be debate about what constitutes a pragmatic trial and how it is distinguished from more traditional explanatory trials. The NIH Pragmatic Trials Collaborative Project, which includes five trials and a coordinating unit, has adopted the Pragmatic-Explanatory Continuum Indicator Summary (PRECIS-2) instrument. The purpose of the study was to collect PRECIS-2 ratings at two points in time to assess whether the tool was sensitive to change in trial design, and to explore with investigators the rationale for rating shifts.

**Methods:**

A mixed-methods design included sequential collection and analysis of quantitative data (PRECIS-2 ratings) and qualitative data. Ratings were collected at two annual, in-person project meetings, and subsequent interviews conducted with investigators were recorded, transcribed, and coded using NVivo 11 Pro for Windows. Rating shifts were coded as either (1) actual change (reflects a change in procedure or protocol), (2) primarily a rating shift reflecting rater variability, or (3) themes that reflect important concepts about the tool and/or pragmatic trial design.

**Results:**

Based on PRECIS-2 ratings, each trial was highly pragmatic at the planning phase and remained so 1 year later in the early phases of trial implementation. Over half of the 45 paired ratings for the nine PRECIS-2 domains indicated a rating change from Time 1 to Time 2 (*N* = 24, 53%). Of the 24 rating changes, only three represented a true change in the design of the trial. Analysis of rationales for rating shifts identified critical themes associated with the tool or pragmatic trial design more generally. Each trial contributed one or more relevant comments, with Eligibility, Flexibility of Adherence, and Follow-up each accounting for more than one.

**Conclusions:**

PRECIS-2 has proved useful for “framing the conversation” about trial design among members of the Pragmatic Trials Collaborative Project. Our findings suggest that design elements assessed by the PRECIS-2 tool may represent mostly stable decisions. Overall, there has been a positive response to using PRECIS-2 to guide conversations around trial design, and the project’s focus on the use of the tool by this group of early adopters has provided valuable feedback to inform future trainings on the tool.

## Background

The need for trials with greater applicability or external validity has been highlighted by many [1–3], and is the most frequent criticism by clinicians of randomized control trials (RCTs), systematic reviews, and guidelines [1]. It has been over 50 years since Schwartz and Lellouch introduced the concept of pragmatic trials, which are performed under more typical conditions with the intention of providing practical results more applicable to clinical practice and decision-making [4, 5]. The design of a pragmatic or effectiveness trial should include a research question relevant to the general population of people with the targeted condition; test interventions in settings close to usual care; and provide more applicable information vital to enabling patients, clinicians, and policymakers to make informed decisions about healthcare. While explanatory (efficacy) trials conducted under ideal conditions play an important role in scientific discovery, healthcare interventions are seldom delivered or monitored under circumstances similar to more constrained trials [6, 7], underscoring the need for pragmatic trials as well.

Despite an exponential rise in the number of trials described as pragmatic, and the creation in 2014 of a new Medical Subject Heading term “pragmatic clinical trial” by the National Library of Medicine (NLM) at the National Institutes of Health (NIH) [8], there continues to be debate about what constitutes a pragmatic trial, how it is distinguished from more traditional explanatory trials, and strategies or tools for designing and describing pragmatic trial characteristics. As there is seldom a purely explanatory or entirely pragmatic trial, there is value in exploring the distinctions underlying this continuum of design decisions, as well as implications of these decisions for the conduct of research (e.g., resources, feasibility, organizational or system buy-in, stakeholder engagement, technological requirements). The Pragmatic-Explanatory Continuum Indicator Summary (PRECIS-2) instrument helps researchers design trials that focus on the end user(s) of trial results and the match between trial design and usual care [9]. PRECIS-2 takes the innovative approach of translating ratings on domains related to trial design to a readily understood wheel format that communicates where the trial design falls on the explanatory-pragmatic continuum. Moher et al.’s review on the value of biomedical research also referenced PRECIS-2 as a tool to help reduce research waste by increasing efficiencies in trial design [10], consistent with the purpose of the tool to assist in planning trials that more closely match the goals of the study. PRECIS-2 was also used by Johnson et al. [11] to indicate trial design decisions for the NIH Health Care Systems Research Collaboratory, which supports pragmatic clinical trial demonstration projects and seeks to create a sustainable infrastructure to improve the design, conduct, and execution of clinical trials. Our project presumes that a secondary benefit of an increase in familiarity with and adoption of the tool is the building of a shared vocabulary for clinical investigators to communicate about trial design decisions and the factors that influence them. In a similar vein, for example, the PRECIS-2 domains were the organizing framework to describe strengths and limitations, and to consolidate the pragmatic features of eight exemplar, large, simple trials [12].

### NIH Pragmatic Trials Collaborative Project

A new effort to implement and learn from a group of pragmatic trials was initiated in 2014 in response to an NIH Request for Applications (RFA) to fund low-cost, pragmatic, patient-centered randomized controlled clinical trials. As specified in the RFA, trials were required to have a minimal, separate research infrastructure and include randomization at the point of patient care, have minimal eligibility criteria, and integrate data collection into or obtained from routine clinical records or existing electronic resources. Interventions were to be delivered as part of routine usual care, with outcomes important to patients and providers. As several NIH institutes were participating in this initiative, variability in interventions and outcomes between the trials was expected. The first phase of the two-phase cooperative agreement funding mechanism, which aims for early identification of trials at risk and funds the UH3 4-year implementation phase contingent on administrative review of milestone achievement, supported start-up activities including refinement of existing resources, further development of study partnerships, conduct of feasibility studies, and finalization of trial protocols. Five of six trials receiving UH2 funds demonstrated trial feasibility and transitioned to the 4-year UH3 trial phase; see Table 1.

**Table 1:** Summary of trials in the Pragmatic Trials Collaborative Project

Trial name	PI/affiliation/sponsor	Trial title	Significance	Setting/target population	Recruitment strategy	Intervention	Primary and (secondary) outcomes	Design/analysis (sample size)
ENGAGES	M. AVIDAN, MD (Washington University)/NIA	Electroencephalograph Guidance of Anesthesia to Alleviate Geriatric Syndromes	Reduce postoperative delirium associated w/cognitive impairment and falls	Hospital/elective surgery patients age 60 + years	Drawn from patients enrolled in SATISFY-SOS study (consent obtained by RA in pre-op clinic)	EEG-guided anesthesia vs. usual care	Postoperative delirium (patient-reported health-related quality of life; postoperative falls)	Block randomization (patients) Intent-to-treat (*N* = 1232)
HUSH	D. BUYSSE, MD (University of Pittsburgh)/NHLBI	Pragmatic Trial of Behavioral Interventions for Insomnia in Hypertensive Patients	Reduce insomnia disorder using non-drug treatment in primary care	Primary care/adult patients w/HTN, hypnotic medication, or insomnia diagnosis	PCP referral via Research Recruitment Alert (RRA); telephone screen; electronic consent	Two CBT interventions (one online) vs. usual care	Self-reported sleep; health indicators (symptoms, health, and patient/provider satisfaction; sleep, depression, anxiety, fatigue)	Stratified block randomization (age and sex) Intent-to-treat (*N* = 625)
PART	H. WANG, MD (University of Alabama at Birmingham)/NHLBI	Pragmatic Trial of Airway Management in Out-of-Hospital Cardiac Arrest	Identification of best approach for out-of-hospital cardio-pulmonary arrest	Community-emergency/non-trauma cardiac arrest – adult patients	N/A	Endotracheal intubation and supraglottic airways approaches	72-hour hospital survival (return of spontaneous circulation, airway management performance, clinical adverse events)	Cluster-crossover (randomization at EMS level – no consent) Intent-to-treat (*N* = 2612)
PROOF Check	M. GONG, MD; O. GAJIC, MD (Albert Einstein College of Medicine of Yeshiva University)/NHLBI	Prevention of Severe Acute Respiratory Failure in Patients w/PROOFCheck	Prevent acute respiratory failure leading to organ failure	Hospital/all at-risk patients	High-risk patients identified by APPROVE (EMR-based)	Clinician notification of high-risk and PROOFCheck (bundle of care practices) vs. usual care	Hospital mortality (organ failure, ventilator-free days, 6- and 12-month mortality, ICU and hospital length of stay, ability to return home on discharge)	Stepped-wedge, cluster randomized (hospital level – no consent) Intent-to-treat (*N *= 7778 minimum)
REDAPS	S. HALPERN, MD (University of Pennsylvania)/NIA	Default Palliative Care Consultation for Seriously Ill Hospitalized Patients	Determine effectiveness and cost of inpatient palliative care consult services	Hospital (w/integrated EHR) Patients ≥ 65 years w/end stage renal disease, advanced COPD, or advanced dementia	Intake assessment (nurse); EHR algorithm generates default palliative care order	Opt-out default for palliative care services vs. usual care (opt-in)	Composite measure hospital mortality and length of stay (hospital and ICU mortality; pain, transfer to ICU and CPR after randomization; days of mechanical ventilation; discharge disposition; 30-day hospital readmission; total hospital costs)	Stepped-wedge, cluster randomized (waiver of informed consent) Intent-to-treat (*N* ≥ 23,000)

*COPD* chronic obstructive pulmonary disease, *CPR* cardiopulmonary resuscitation*, EEG* electroencephalogram, *EHR* electronic health record, *EMR* electronic medical record*, HTN* hypertension, *ICU* intensive care unit, *NIA* National Institute on Aging, *N/A* not applicable, *w/*with

Trials: *ENGAGES* Electroencephalograph Guidance of Anesthesia to Alleviate Geriatric Syndromes Trial, *HUSH* Pragmatic Trial of Behavioral Interventions for Insomnia in Hypertensive Patients, *PART* Pragmatic Trial of Airway Management in Out-of-Hospital Cardiac Arrest*, PROOFCheck* Prevention of Severe Acute Respiratory Failure in Patients w/PROOFCheck (Electronic Checklist to Prevent Organ Failure), *REDAPS* Default Palliative Care Consultation for Seriously Ill Hospitalized Patients

A separate award established a coordinating unit to support collaborative activities and monitor milestone achievement. Awardees and NIH project officers participate in joint activities to gain a better understanding of the struggles and successes of trial planning and implementation, explore the significance of stakeholder engagement to trial success, and identify challenges to meeting recruitment and retention goals.

### Objectives

The purpose of this study was to collect PRECIS-2 ratings at two points in time, assess whether the tool was sensitive to change in design, and explore investigators’ experiences and impressions of the tool. In this paper, we describe how the tool was introduced and used by members of the Pragmatic Trials Collaborative Project, and synthesize findings from discussions with the trial investigators to further clarify important distinctions pertaining to the explanatory-pragmatic continuum.

### The PRECIS-2 tool

The tool is designed to assess and document the position of a trial within the pragmatic-explanatory continuum [13–16]. PRECIS-2 requires investigators to consider nine distinct domains in relation to the intended purpose of the trial; these can be rated from “1” ideal setting (explanatory) to “5” more real-world, usual care (pragmatic). The domains covered include *Eligibility Criteria*, *Recruitment Path*, *Setting, Organization, Flexibility of Delivery of Experimental Intervention, Flexibility of Adherence of Experimental Intervention, Follow-up, Primary Outcome,* and *Primary Analysis* (see Table 2). Evidence from the interactive PRECIS-2 website resource (https://www.precis-2.org/) [17] indicates that the tool is being used by investigators across many different contexts [18–25], and that wheel results are included in study protocols [26, 27].

**Table 2:** Nine PRECIS-2 domains for assessing trial designing characteristics^a^

Domain	Assessment considerations
Eligibility	To what extent are the participants in the trial similar to patients who would receive this intervention if it was part of usual care? For example, score 5 for very pragmatic criteria essentially identical to those in usual care; score 1 for a very explanatory approach with lots of exclusions (e.g., those who do not comply, respond to treatment, or are not at high risk for primary outcome, are children or elderly), or uses many selection tests not used in usual care
Recruitment Path	How much extra effort is made to recruit participants over and above what would be used in the usual care setting to engage with patients? For example, score 5 for very pragmatic recruitment through usual appointments or clinic; score 1 for a very explanatory approach with targeted invitation letters, advertising in newspapers, radio plus incentives and other routes that would not be used in usual care.
Setting	How different are the settings of the trial from the usual care setting? For example, score 5 for a very pragmatic choice using identical settings to usual care; score 1, for a very explanatory approach with only a single center, or only specialized trial or academic centers^b^
Organization	How different are the resources, provider expertise, and organization of care delivery in the intervention group of the trial from those available in usual care? For example, score 5 for a very pragmatic choice that uses identical organization to usual care; score 1 for a very explanatory approach if the trial increases staff levels, gives additional training, require more than usual experience or certification and increase resources
Flexibility in Delivery	How different is the flexibility in how the intervention is delivered from the flexibility anticipated in usual care? For example, score 5 for a very pragmatic choice with identical flexibility to usual care; score 1 for a very explanatory approach if there is a strict protocol, monitoring and measures to improve compliance, with specific advice on allowed co-interventions and complications
Flexibility in Adherence	How different is the flexibility in how participants are monitored and encouraged to adhere to the intervention from the flexibility anticipated in usual care? For example, score 5 for a very pragmatic choice involving no more than usual encouragement to adhere to the intervention; score 1 for a very explanatory approach that involves exclusion based on adherence, and measures to improve adherence if found wanting. In some trials e.g., surgical trials where patients are being operated on or intensive care unit trials where patients are being given intravenously administered drug therapy, this domain is not applicable as there is no compliance issue after consent has been given, so this score should be left blank
Follow-up	How different is the intensity of measurement and the follow-up of participants in the trial from the typical follow-up in usual care? For example, score 5 for a very pragmatic approach with no more than usual follow-up; score 1 for a very explanatory approach with more frequent, longer visits, unscheduled visits triggered by primary outcome event or intervening event, and more extensive data collection
Primary Outcome	To what extent is the primary outcome of the trial directly relevant to participants? For example, score 5 for a very pragmatic choice where the outcome is of obvious importance to participants; score 1 for a very explanatory approach using a surrogate, physiological outcome, central adjudication or use assessment expertise that is not available in usual care, or the outcome is measured at an earlier time than in usual care
Primary Analysis	To what extent are all data included in the analysis of the primary outcome? For example, score 5 for a very pragmatic approach using intention-to-treat with all available data; score 1 for a very explanatory analysis that excludes ineligible post-randomization participants, includes only completers or those following the treatment protocol

^a^PRECIS-2. 2016. https://www.precis-2.org/ [17]

^b^Instructions to rate Setting are derived from a systematic review done with physicians in Toronto on the hypertension trials. The new scheme addresses the question of *How different are the settings of the trial from the usual care setting?*

5 = Trial is multi-center and all centers are typical of those for treating patients with hypertension in usual care

4 = Trial is multi-center but one or two of the centers are not usual care but specialized settings e.g., lead center university or specialized secondary care are also centers

3 = Trial is multi-center but many of the centers appear not typical of usual care

2 = A single center which may be similar to usual care setting for treating patients with hypertension. Even if it is a primary care center

1 = A single center definitely specialized or academic center not typical of usual care setting for patients with hypertension

## Methods

### Study participants

The five principal investigators (PIs) funded under this initiative participated in the study. All members of the project in attendance (which includes the coordinating unit staff and nine NIH program staff officers) participated in discussions and training on the PRECIS-2 tool and in development of the concept for this paper. The PIs had the opportunity to provide feedback on an early draft of the findings.

Our mixed-methods design structure can be described as quantitative analysis preceding qualitative analysis (quan → QUAL)—i.e., sequential collection and analysis of quantitative data (PRECIS-2 ratings) and qualitative methods, specifically follow-up interviews with PIs, with emphasis on the qualitative data. The function of the analysis was primarily expansion, whereby the qualitative data were used to understand what shifts in PI ratings reflected about the pragmatic trial domains [28, 29]. PRECIS-2 ratings were recorded on a worksheet and collected at two annual, in-person project meetings. After a presentation and brief training on the tool at the first meeting shortly after funding awards for the planning phase were made (February 2015; Time 1), PIs rated their trials as currently designed based on their knowledge of the trial. Following a refresher session at the second annual meeting shortly after trials were initiated (April 2016; Time 2), PIs again rated the current status of their trials on each domain, without reference to Time 1 ratings or to any other documentation. Subsequently, a semistructured interview guide was developed to frame the conduct of qualitative telephone interviews, conducted with each PI by author PDL in summer 2016. In addition to discussing domains with a change in ratings, PIs were asked about their impressions of the tool (*Have you used the tool when designing other trials*?; *Have you recommended the tool to colleagues or seen an increase in use of the tool*?; and *Do you have any other feedback regarding the tool*?) and to provide ratings as follows: *How strongly do you agree with these two statements, on a rating scale from 5 (strongly agree) to 1 (strongly disagree)?* [16]; (1) PRECIS-2 would have been useful in the design phase of the trial and (2) PRECIS-2 highlighted areas of trial design which are important for your trial to achieve its goals.

A summary table of PI ratings was provided in advance of the call for reference during the discussion. For each rating change, by domain, the interviewer noted the direction of the change (more or less pragmatic), and asked the PI what changed from Time 1 (T1) to Time 2 (T2). If the revised rating truly reflected a modification to the trial, the PI provided a description and rationale. If there was no trial modification, the PI was asked to explain the rating shift. The focus of the discussion was on detecting trial design change and not on the value or direction of the rating. Interviews were recorded and transcribed and coded using NVivo version 11 Pro for Windows, Baltimore, US licensed issued from October 2016 through 2017. The documents were initially coded by one team member (DM), who used NVivo to extract each instance of PRECIS-2 rating shifts and content to analyze the stated circumstances and rationale. The results of the initial coding and analysis were reviewed and deliberated in depth with team members (KL, RM, DM, PDL, LD) to agree on the characterization of each instance. From this analysis, four categories of rating changes were initially identified which included two separate categories labeled “miscategorization” and “misunderstanding.” However, when the analysts independently coded the themes there was lack of inter-coder reliability suggesting that these were indistinct. The final analysis included: (1) actual change (reflects a change in procedure or protocol), (2) primarily a rating shift reflecting rater variability (e.g., the PIs rating changed but not due to a trial adaptation), and (3) themes that reflect important concepts about the tool and/or pragmatic trial design (further explored for additional clarity regarding use of the tool).

## Results

### PRECIS-2 ratings

**Fig. 1:**
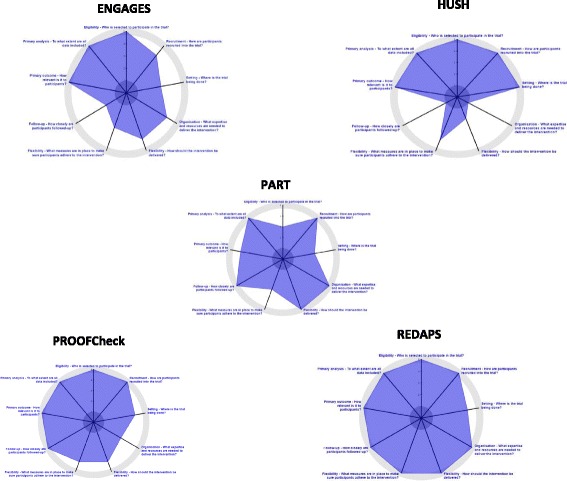
PRECIS-2 principal investigator (PI) plots by study trial (trial planning phase – Time 1)

PI ratings at each time point (T1, T2) are presented in Table 3 below. T1 ratings were used to generate a trial-specific PRECIS-2 plot using the tool on the PRECIS-2 website (https://precis-2.org/) [17]; see Fig. 1 below.

**Table 3:**
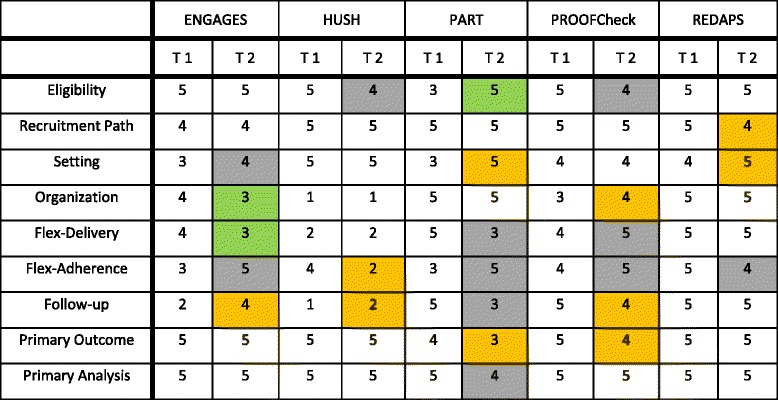
PRECIS-2 principal investigator (PI) ratings at trial planning (Time 1) and trial implementation (Time 2)

Actual change (*N* = 3, 13%)

Rating shift/rater variability (*N* = 10, 42%)

Thematic responses requiring clarification (*N* = 11, 46%)

When assessing whether the trials met the RFA requirements that aligned with the PRECIS-2 domains (specifically, Eligibility, Recruitment Path, Organization, Flexibility of Intervention Delivery, Follow-up, and Primary Outcomes) we found that all but one was rated toward pragmatic (ratings of 4 or 5) on five of six domains (for the first rating T1 reflecting the UH2 planning phase). Ratings lower than 4 were found for the Electroencephalograph Guidance of Anesthesia to Alleviate Geriatric Syndromes (ENGAGES) trial [30] (Follow-up), for the Pragmatic Trial of Behavioral Interventions for Insomnia in Hypertensive Patients (HUSH) trial [31] (Organization, Flexibility of Intervention Delivery, Follow-up), for the Pragmatic Trial of Airway Management in Out-of-Hospital Cardiac Arrest (PART) trial [32] (Eligibility), and for the Prevention of Severe Acute Respiratory Failure in Patients (PROOFCheck) trial [33] (Organization). The five PIs completed paired ratings (i.e., T1 to T2) for the nine PRECIS-2 domains) for a total of 45 paired ratings. Those indicating a rating change from T1 to T2 (*N* = 24, 53%) were the focus of the qualitative data collection and analysis.

### Qualitative findings

Three broad categories of responses were identified. Of the 24 rating changes, only three represented a true change in the design of the trial. The remaining responses were evenly split between simple rating shifts reflecting rater variability (*N* = 10), and those reflecting important concepts about the tool or pragmatic trial design (*N* = 11).

#### Change in trial design

For the PART trial [32], loosening of eligibility criteria over time resulted in a design shift toward pragmatism. Initially, there were more exclusion criteria, but the PI indicated that several criteria were removed as of the second rating period (implementation). The final rating on the Eligibility domain was a “5,” reflecting that trial participants were perceived to be nearly identical to those likely to receive the intervention under usual care conditions: *“We did loosen up one or two more additional criteria,” stated the PART PI, “[5 is the correct rating because] we are including as broad a selection of patients as possible with very few exclusion criteria.”*

The ENGAGES trial [30] experienced two design changes in the more explanatory direction. The PI explained that they “*discovered that we do actually need more training [of the clinicians] than anticipated so that’s why I think Organization is a bit more explanatory than we had…anticipated originally.”* Because additional organizational resources to train the clinicians were needed (beyond those available in usual care) the rating on this domain shifted to a less pragmatic rating. The need to increase monitoring of intervention delivery contributed to a shift in rating for the Flexibility of Intervention Delivery domain. As described by the PI, *“I changed [the rating] because the protocol is a little bit more prescriptive than initially suggested. A [lower score] is reasonable considering our experience now. I think that a 4 is what we had anticipated and a 3 is closer to our actual experience.”*

#### Shift in rating

Examples of statements primarily reflecting rater variability included the following: (1) *“I don’t think it’s become less pragmatic [Recruitment Path]. I think I probably overrated its pragmatism the first time,”* and (2) *“I’m not sure why I gave it a 4 the first time. I mean it’s about as pragmatic as it gets [Setting].”* In some cases the shift was attributed to a new understanding of the trial rather than a change in design. For example, with regard to Setting, one investigator concluded that the study sites are more representative and comparable to usual care than was initially thought, and another realized that intensity of Follow-up was less than anticipated and did not involve additional patient visits.

#### Pragmatic trial themes

The remaining responses are the primary focus of the analysis, as they illuminated important concepts relevant to either the tool or to the design of pragmatic trials. Each trial contributed one or more comments, which fell within six of the nine domains, with Eligibility, Flexibility of Adherence, and Follow-up each accounting for more than one. Issues (by domain), the associated PI statement, and clarification regarding the rationale for the rating are presented in Table 4.

**Table 4:** Thematic responses and clarification by domain (*N* = 11)

Domain (*N*)	Interpretation and PI responses	Domain clarification
Eligibility	1. Less pragmatic due to additional effort needed to identify appropriate patients and to validate correct identification: *“It’s a little more work to figure out patients who are chronically vented who are excluded…I just thought it was going to be very, very easy and you don’t have to think about it. But it turns out actually I have to have my staff validate it.”* (PI)	Eligibility refers to the extent to which the trial population matches the population intended for the intervention. The issue of effort to engage participants is more relevant to Recruitment Path, which addresses whether effort to recruit participants is greater than for patient engagement in usual care
	2. Less pragmatic due to a higher proportion of patients excluded than originally anticipated: *“Once we started applying the criteria, we recognized…there are some people who we’ve excluded and I think they’re for good reasons… we haven’t changed the criteria, it’s just that as we’ve been applying them, we realized that it excludes a larger percentage of people perhaps than we thought.”* (PI)	For the Eligibility domain one should consider the extent to which trial participants are similar to those who would receive the intervention if it were part of usual care (rather than volume of participants excluded)
Setting	3. Setting is similar to usual care. *“The setting is really the identical setting to usual care. But I probably scored it a bit down [at T1] it’s very representative of a usual setting.”* (PI)	Setting receives a more explanatory score if there is only a single center, or only a specialized trial or academic center. Multi-center trials can be rated 3–5
Flexibility-Delivery	4. More pragmatic because clinician notification (re: eligibility) was more automated than anticipated. *“When we were in the planning phase…not clear how we were going to notify the [___]. It turns out the hospital itself had an outside vendor trying to figure out actually how to link …that [mechanism] is part of the hospital infrastructure now.”* (PI)	Resource requirements are addressed under the Organization; the issue of resources required to conduct the study is not relevant to intervention delivery or adherence
Flexibility-Adherence	5. More pragmatic because notification (re: patient eligibility) was more automated than anticipated^a^: *“When we were in the planning phase, [it wasn’t] clear to us exactly how we were going to notify the ___ – ”* (PI)	Resource requirements are addressed under the Organization (see above). This domain should not have been rated as there is no monitoring of patient adherence
	6. More pragmatic because no participants are excluded due to adherence: *“We’re not excluding anybody based on adherence, but we are encouraging adherence and are providing feedback on adherence.”* (PI)	This domain addresses how flexibly participants in the trial are monitored and encouraged compared to usual care. This domain is not applicable to 2 of the trials as there is no compliance issue after consent has been given. The domain should be left blank (unrated)
	7. More pragmatic because the intervention is executed in emergency care and adherence is minimal: *“Our intervention really is executed and then it’s done, so the adherence of it is actually very minimal and the remainder of care given thereafter is just standard of care.”* (PI)	
	8. Less pragmatic because there is no usual care comparison: *“There’s no way to know what would happen in usual care because the intervention’s never been tried in usual care. But I would foresee if our results prove favorable that the implementation in the real world would be identical to what we’re testing.”* (PI)	The issue of usual care comparison is relevant to Flexibility of Intervention Delivery rather than Adherence
Follow-up	9. Less pragmatic as collection of follow-up requires more effort than anticipated: *“In clinical care, one would not necessarily seek out follow-up on patients,…what made us think that it was less pragmatic was the manner by which you seek out that information.”* (PI)	Does not apply to this domain, which is concerned only with burden of follow-up on the participants, not whether the follow-up data are routinely collected
	10. Less pragmatic as collection of follow-up is less automatic than anticipated: *“I have to apply in a separate IRB to a statistics department to get that long term follow-up. And that requires linking of the patient’s data. So that’s just a little less automatic…more work for me. For patients it’s the same.”* (PI)	This domain is concerned only with burden of follow-up on the participants, not burden on research team or effort needed to collect the follow-up data
Primary Analysis	11. Less pragmatic because the primary outcomes are not a standard measure: *“The analysis is a standard analysis one would do for this type of a trial, but [not] a standard comparison that one would make on a daily basis.”* (PI)	Pragmatism of primary analysis is based only on the degree to which all data are included in the analysis of the primary outcome

^a^Same consideration was applied for both Flexibility of Delivery and Flexibility of Adherence

### Reflections on the tool

Two of the PIs had used the original PRECIS (2009) [14] tool to assess the design of their protocols. The other three were unfamiliar with the tool (or its predecessor) prior to the project; however, all agreed that PRECIS-2 would have been helpful at the design phase of their trials (Table 5).

**Table 5:** Principal investigator (PI) reflections on the PRECIS-2 tool

Rating scale	Would have been useful in design phase (*N*)	Highlighted areas important for trial to achieve goals (*N*)
Strongly agree	2	1
Somewhat agree	3	2
Neither agree or disagree	0	1
Somewhat disagree	0	1
Strongly disagree	0	0

One of the PIs reported recently using the tool in a grant application, and two others reported disseminating it to other colleagues, *“[I am] continuing to use it. And we have recommended it to colleagues.”* Another PI commented that the tool would *“be useful for myself and others in designing future studies”* stating *“I really do hope that some of my colleagues start to use it on a more routine basis [to] report…just like clinical trials have to include a CONSORT flow chart.”* Other illustrative positive statements indicated that the tool was helpful as a *“good mental/academic exercise”* and that *“it does help me conceptualize my argument about why my trial is pragmatic.”*

The respondents also reflected lingering confusion regarding the tool: *“I think that even now some of them [domains] are very obvious and intuitive…some of them the ranges in the scores were very tight and some the ranges were quite wide and I think that reflects some ambiguity in the tool.”* There were also several comments pertaining to specific domains. For example, *“…Eligibility and Recruitment are really…key…[to] how we design trials…and its guided our thinking in terms of the effects of different consent approaches, which are appropriate for pragmatic trials*.” An issue with the Follow-up domain was described thus: *“We’d like to get long-term outcomes data, but that would really change the budget requirements and the pragmatism considerably.”*

## Discussion

PRECIS-2 is a useful tool that increases transparency in design decisions and which has proved useful for “framing the conversation” about trial design among members of the Pragmatic Trials Collaborative Project. This concurs with experiences at the Pragmatic Clinical Trials Unit in London where the PRECIS-2 tool highlighted trial design decisions, which facilitated valuable discussion [16]. Based on PRECIS-2 ratings, each of the five trials was highly pragmatic at the planning phase and remained so 1 year later in the early phases of trial implementation. Our approach to using the tool at different points in time to detect change suggests that the design elements assessed by the PRECIS-2 tool may represent mostly stable decisions. Using this methodology, we identified only two trials with any actual changes in design.

Discussions with the PIs also helped to identify several issues that are important to address as we refine the conversation around the use of PRECIS-2, seek to improve the tool, and advance our understanding of pragmatic design decisions. The remainder of our discussion draws on specific information from the trials to further elaborate on these elements in relation to the domain ratings.

### Eligibility

This domain can have a range of ratings if there is uncertainty about who would be treated in usual care for a particular condition. It requires in-depth knowledge about the research area and can be one of the most common areas reducing external validity of results [6, 7]. A pragmatic trial would include anyone who would usually receive the intervention, and exclude those who would not routinely get the treatment (regardless of the number of people in this group):

- For example, in the PART trial [32] testing airway management for resuscitation from out-of-hospital cardiopulmonary arrest (OHCA) endotracheal intubation (ETI) supraglottic airways (SGA), participants included were all adults who needed airway management following cardiac arrest. Exclusions were vulnerable populations who had “Do Not Resuscitate” orders, traumatic cardiac arrest, and children, which are routine exclusions and, therefore, very pragmatic. The only group that would be treated in usual care that were excluded were pregnant women and prisoners but these are usual Institutional Review Board (IRB) exclusions as protected groups.

### Organization

There can be ambiguity in scoring the Organization domain as this includes knowledge of the current organization including existing healthcare staff and resources. Adding additional resources or infrastructure solely for the purposes of research (i.e., not part of the intervention) moves the rating more in the explanatory direction:

- For example, in the HUSH trial [31], cognitive behavioral therapy (CBT) delivery for insomnia was being tested in primary care using three methods requiring different resources. Thus, the Organization domain should have been rated separately for the two interventions being compared to usual care. One (Sleep Healthy Using the Internet) included a self-guided Internet version of CBT and the second (Brief Behavioral Treatment of Insomnia) involved a provider who would likely need additional training.

### Flexibility of Intervention Adherence

Each trial had a rating change on this domain, suggesting that it may be harder to assess initially, in particular when considering recipients who have consented to a procedure but thereafter are not involved in adherence issues with the intervention. This domain can benefit from discussion to create consensus among trial designers due to uncertainty in how much monitoring and feedback is routine; a fully pragmatic design would accept full flexibility in how the patient (recipient) interacted with the intervention. Several of the trials illustrate challenges with rating this domain:

- In the ENGAGES trial [30], testing if an electroencephalography-guided protocol in elderly patients undergoing major elective surgery decreases the incidence of postoperative delirium, it could be argued that this domain was not applicable as the patients had given consent to the operation and being part of the trial. The official guidance [9] in the PRECIS-2 toolkit is that the domain should not be rated, as follows, *“In some trials, e.g., surgical trials where patients are being operated on or intensive care unit trials where patients are being given intravenously administered drug therapy, this domain is not applicable as there is no compliance issue after consent has been given, so this score should be left blank.”*
- Similarly, in the PART trial [32] this domain is also not applicable as there was no compliance from the patient who either got ETI or SGA airway management resuscitation for OHCA.
- For the PROOFCheck trial [33] the domain is also not applicable as patients were not involved in compliance; confusion may have occurred because adherence for physicians (the interventionist) was tested on use of checklists to determine which patients required mechanical ventilation to prevent severe acute respiratory failure (ARF). This domain is relevant for the HUSH trial [31]. For patients in the SHUTi arm of the trial adherence was encouraged using automated emails only, whereas in the Brief Behavioral Treatment of Insomnia (BBTI) arm the provider reviews progress with the participant for 15–30 minutes each week for 3 weeks to adjust sleep/wake times, which might be related to usual encouragement from a doctor. Due to the different ways of encouraging adherence, however, each of these interventions may be rated differently by trialists. Further clarification may be needed to assist trialists to score this domain accurately. In the REDAPS trial [34], however, with an intervention to test out the default option for palliative care consultation, the PI anticipated that the intervention could be fully pragmatic “5” but had marked down to “4” due to uncertainty implementing into usual care.

### Follow-up

Burden on the research team to collect the follow-up data required to address the primary research question is not assessed on the PRECIS-2 tool and was misapplied to Follow-up in two instances. The only consideration for this domain is how different is the intensity of measurement and follow-up for patients/participants from what is typical in usual care. The effort required to collect follow-up data is also not a consideration under Organization, which pertains to the resources required to deliver the intervention, not the effort to measure outcomes.

## Conclusions

Use of the PRECIS-2 tool has provided an important framework for the project team to organize observations about critical elements underlying design decisions that impact the position of the trial along the pragmatic-explanatory continuum, and to communicate more effectively with their trial stakeholders about these elements. Prior to the Pragmatic Trials Collaborative Project, two of the PIs were familiar with the earlier version of the tool, and none had had extensive training or experience applying it to trial design. The training provided at each annual meeting was less extensive than trainings intended to achieve inter-rater reliability; therefore, it is not unexpected that the rationales provided to justify ratings revealed several common themes requiring additional clarification. Continuing to refine our understanding, we believe, is critical for communicating about decisions and for valid comparisons of design characteristics and their consequences.

What we have learned from ongoing monitoring of milestone achievement during the planning phase is that these low-cost trials required sustained attention to a range of underlying shifts in healthcare delivery and health system operations. These scenarios can create surprise challenges for investigators well into the pragmatic clinical trial life span. Our conclusion that trial design decisions may be relatively stable, even for pragmatic trials subject to real-world implementation challenges, should be further explored in a larger set of pragmatic trials. Furthermore, we did not explore trial changes not reflected in the PRECIS domain structure, discuss domains with no change in rating as they were beyond the scope of our qualitative follow-up, or check for false negatives wherein actual domain changes were not captured.

Our Pragmatic Trials Collaborative Project is a timely opportunity to understand the contexts in which complex pragmatic trials are being conducted, and the investigators and NIH project officers have benefitted from learning how each study team is striving to ensure that they fulfill the intended purpose of the trial. Overall, there has been a positive response to using PRECIS-2 to guide conversations around trial design, and the project’s focus on the use of the tool by this group of early adopters has provided valuable feedback to inform future trainings on the tool. In addition to evidence that the tool is increasingly included in study protocols and publications, the use of the tool in proposals indicates a critical need for sponsors of pragmatic trials and members of review panels—as well as future trial designers—to be knowledgeable regarding how to rate and interpret the PRECIS-2 ratings.

## Abbreviations

ARF: Acute respiratory failure
BBTI: Brief Behavioral Treatment of Insomnia
CBT: Cognitive behavioral therapy
ENGAGES: Electroencephalograph Guidance of Anesthesia to Alleviate Geriatric Syndromes Trial
ETI: Endotracheal intubation
HUSH: Pragmatic Trial of Behavioral Interventions for Insomnia in Hypertensive Patients
IRB: Institutional Review Board
NHLBI: National Heart, Lung, and Blood Institute
NIA: National Institute on Aging
NIH: National Institutes of Health
NLM: National Library of Medicine
OHCA: Out-of-hospital cardiopulmonary arrest
PART: Pragmatic Trial of Airway Management in Out-of-Hospital Cardiac Arrest
PI: Principal investigator
PRECIS-2: Pragmatic-Explanatory Continuum Indicator Summary
PROOFCheck: Prevention of Severe Acute Respiratory Failure in Patients w/PROOFCheck (Electronic Checklist to Prevent Organ Failure)
quan → QUAL: Quantitative analysis preceding qualitative analysis, with qualitative analysis dominant
RCT: Randomized control trials
REDAPS: Default Palliative Care Consultation for Seriously Ill Hospitalized Patients
RFA: Request for Applications
SGA: Supraglottic airways
SHUTi: Sleep Healthy Using the Internet
T1: Time 1 (trial planning phase)
T2: Time 2 (trial implementation phase)
UH2/UH3: NIH activity codes used to support research activities in a specific category to the planning and implementation phases of a trial
